# *Rice black‐streaked dwarf virus*: From multiparty interactions among plant–virus–vector to intermittent epidemics

**DOI:** 10.1111/mpp.12946

**Published:** 2020-06-08

**Authors:** Nan Wu, Lu Zhang, Yingdang Ren, Xifeng Wang

**Affiliations:** ^1^ State Key Laboratory for Biology of Plant Diseases and Insect Pests, Institute of Plant Protection Chinese Academy of Agricultural Sciences Beijing China; ^2^ Institute of Plant Protection Henan Academy of Agricultural Sciences Zhengzhou China

**Keywords:** cereal crops, epidemics, fijiviruses, planthoppers, *Reoviridae*

## Abstract

Rice black‐streaked dwarf virus (RBSDV) (species *Rice black‐streaked dwarf virus*, genus *Fijivirus*, family *Reoviridae*) is the causal agent of rice black‐streaked dwarf and maize rough dwarf diseases, which occur in intermittent epidemics in East Asian countries and are responsible for considerable yield losses. Intermittency of epidemics make accurate forecasting and designing of effective management strategies difficult. However, recent insights into host–virus–vector insect interactions are now informing forecasting and disease control measures. Resistance genes are also being identified and mapped.

**Symptomatology and host range:**

RBSDV induces extreme stunting, darkened, and stiff leaves of crops and weeds only in the family Poaceae, including *Oryza sativa*, *Zea mays*, and *Triticum aestivum*. Infected plants produce totally or partially deformed panicles and remain alive through harvest.

**Genome and gene function:**

The nonenveloped virus particles comprise a double‐layered capsid, 50‐nm core with genomic double‐stranded RNA (dsRNA), and six proteins. The genome of RBSDV contains 10 segments of dsRNA, named S1 to S10 in decreasing order of molecular weight. Segments 1, 2, 3, 4, 6, 8, and 10 encode the RNA‐dependent RNA polymerase (RdRp), the major core structural protein, a protein with guanylyltransferase activity, an outer‐shell B‐spike protein, viral RNA‐silencing suppressor, the major capsid protein, and the outer capsid protein, respectively. Each of the segments 5, 7, and 9 encodes two proteins: P5‐1, a component of viroplasms; P5‐2 of unknown function; nonstructural protein P7‐1, involved in forming the structural matrix of tubular structures in infected tissues; P7‐2 of unknown function; P9‐1, the main component of viroplasms in infected cells and involved in viral replication; and P9‐2 of unknown function.

**Transmission and epidemiology:**

RBSDV is transmitted by *Laodelphax striatellus* in a persistent propagative manner. The vector insect is the only means of virus spread in nature, so its migration and transmission efficiency are obligatory for disease epidemics to develop. Susceptible varieties are widely planted, but efficient transmission by vectors is the primary reason for the epidemics. Cultivation system, pesticide overuse, and climatic conditions also contribute to epidemics by affecting the development of the vector insects and their population dynamics.

**Disease management:**

In the absence of resistant varieties, integrated disease management aims at disrupting the cycle of virus transmission by the insect vector. Inheritance studies have indicated that resistance is mostly governed by quantitative trait loci or multiple genes. Genetic engineering through RNA‐interference and gene‐editing strategies are potential approaches for disease control.

## Graphical abstract

This pathogen profile summarizes the current knowledge on *Rice black‐streaked dwarf virus*, which causes rice black‐streaked dwarf and maize rough dwarf diseases.

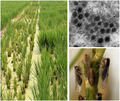

## 1 INTRODUCTION

Staple cereal or grain crops, mainly rice, wheat, and maize, belonging to the grass family *Poaceae*, provide a majority of society's caloric needs throughout the world, but the last few decades have shown that yields, and thus people's nutrition, can be greatly compromised by virus diseases (Suzuki *et al*., 2015). In general, virus diseases of cereals can be divided into those caused by either seed‐ or soilborne viruses or by insect‐transmitted viruses (Slykhuis, 2003). Among the insect‐transmitted viruses, the most important in rice are the planthopper‐transmitted rice stripe virus (RSV) (species *Rice stripe virus*, genus *Tenuivirus*, family *Phenuiviridae*), and rice black‐streaked dwarf virus (RBSDV) and southern rice black‐streaked dwarf virus (SRBSDV) (species *Rice black‐streaked dwarf virus* and *Southern rice black‐streaked dwarf virus*, genus *Fijivirus*, family *Reoviridae*) (Uehara‐Ichiki *et al*., 2013; Liu *et al*., 2018). Here we will focus on RBSDV.

RBSDV was first identified in Japan, where it seems to have existed for many years but was only reported to cause a new rice disease in 1952 (Kuribayashi and Shinkai, 1952). The first major outbreaks of RBSDV in Japan were recorded in maize during 1957–1961 and in rice and maize during 1965–1967 (Hibino, 1996). In 1963, it was reported from Yuyao County, Zhejiang Province in China and in Korea sporadically in limited fields in the form of small patches but causing considerable yield losses during the mid‐1960s. It seemed to have disappeared in eastern China after 1967, and it was hard to find diseased samples in the fields (Chen, 1964; Ou, 1985; Chen and Zhang, 2005; Lee *et al*., 2005). However, RBSDV re‐emerged in hybrid rice fields in Zhejiang Province in China during 1991–2002. Since 2008, outbreaks in Jiangsu, Shandong, and Henan provinces in China have been recorded in japonica rice (Figure S1a,b), maize (Figure S1c), and wheat (Figure S1d) (Ren *et al*., 2016), but in recent years no serious epidemics have occurred (Liu *et al*., 2020, 2020a). RBSDV incidence in rice also began to increase in Saitama Prefecture in Japan and the Jeonra regions in Korea after 2010 (Matsukura *et al*., 2019). Such intermittent outbreaks hinder forecasting and the design of effective disease management strategies.

Many aspects of the disease, including virus physical properties and detection, genome sequences and diversity, transmission characteristics by its insect vector, variety resistance, and disease control, have been studied intensively, but the research literature has not yet been reviewed. This review therefore summarizes the current knowledge on symptoms on different cereal crops, genome organization and gene functions, different diagnostic tests, virus transmission, factors contributing to intermittent outbreaks, the genetics of host resistance, and disease management. We also propose future research directions to better understand the intermittent outbreaks and manage this disease problem.

## 2 SYMPTOMATOLOGY AND HOST RANGE

**Figure 1:**
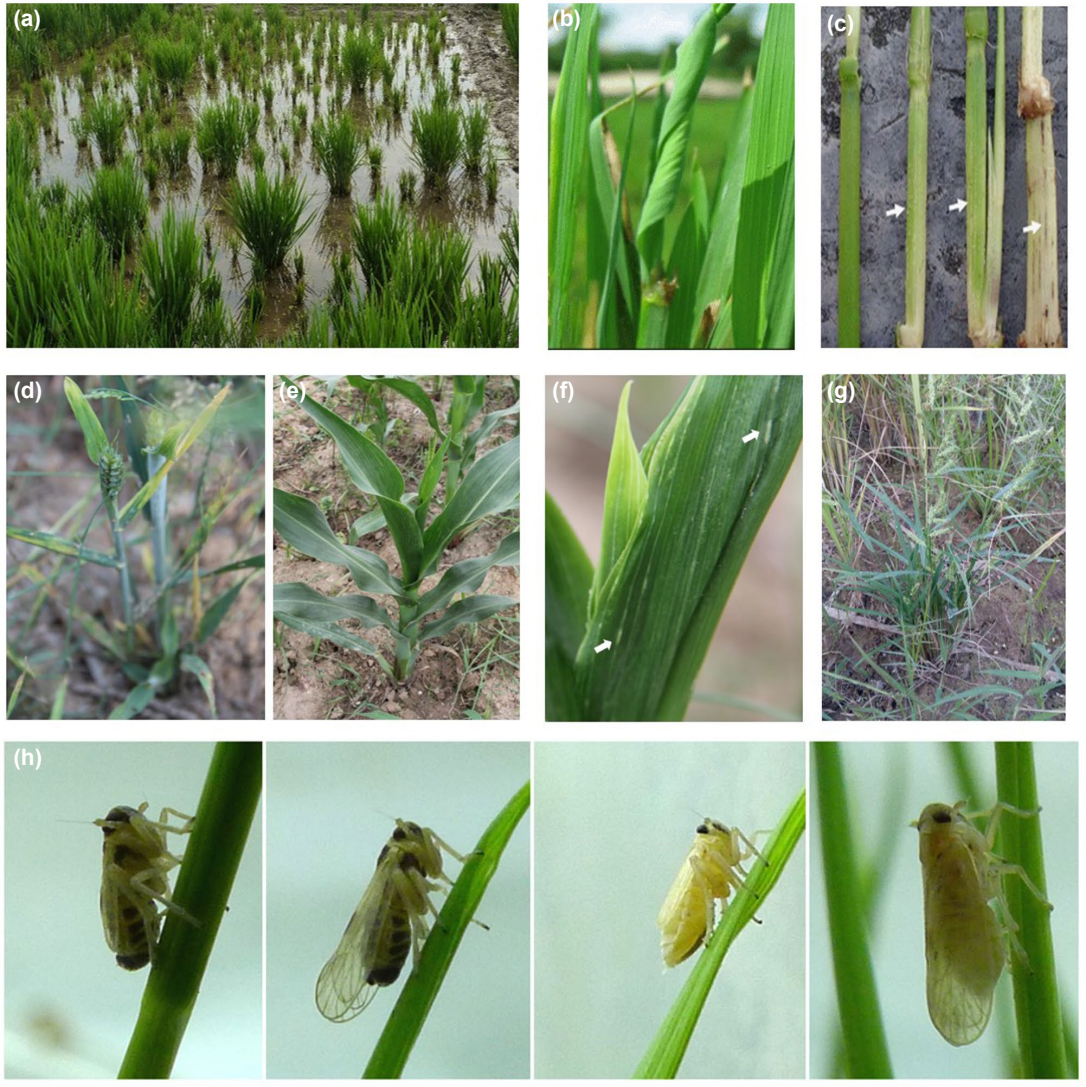
Symptoms of rice black‐streaked dwarf virus (RBSDV)‐induced diseases and the vector *Laodelphax striatellus* (small brown planthopper). (a) Diseased rice plants in field. (b) Wrinkling and twisting of rice leaves. (c) White waxy enations on rice stem. (d) Diseased wheat plants. (e) Diseased maize plants. (f) White waxy enations (tumours) on abaxial veins of maize leaves. (g) Diseased barnyard grass plants. (h) Vector insect on rice stem, from left to right: female nymph, female adult, male nymph, and male adult

In general, most japonica varieties of rice (*Oryza sativa*) are more susceptible to RBSDV than indica varieties (Morinaka and Sakurai, 1968). Susceptible japonica paddy rice usually develops extreme stunting, darkening of leaves, and twisting of the distal portions of young leaves (Figure 1a,b). White waxy enations on veins, lower sides of leaf blades, outer sides of leaf sheath, and the stem are characteristic of RBSDV‐infected plants at a later stage of infection (Figure 1c). The infected plants produce either totally or partially deformed panicles and remain alive through harvest. Infected plants of wheat (*Triticum aestivum*) are severely stunted and produce twisted leaves, sometimes with white, waxy swellings on the veins on the abaxial surfaces of leaves and culms (Figure 1d). Diseased maize (*Zea mays*) plants are dwarfed with darkened leaves (Figure 1e). At an early stage of infection, white streaks appear along the veins, and white, waxy enations occur on the veins on abaxial leaf surfaces, which are also sometimes called “tumours” (Figure 1f). The margins of affected leaves might be split at late stages of infection (Shinkai, 1962). Dwarfed and darkened leaf symptoms were observed on barnyard grass (*Echinochloa crus‐galli*) (Figure 1g).

All known host plants of RBSDV, including rice, maize, wheat, oat (*Avena sativa*), barley (*Hordeum vulgare*), *Alopecours aequalis*, *Beckmannia syzinachne*, *Poa annua*, and some other gramineous weeds (Shikata, 1974), are members of the grass family Poaceae, which can be infested with high density with the small brown planthopper (SBPH, *Laodelphax striatellus*) (Figure 1h). A recent study showed that RBSDV can complete its infection cycle in Italian ryegrass (*Lolium multiflorum*). In addition, *Avena fatua*, *Avena sterilis* subsp. *ludoviciana*, *Cynosurus echinatus*, *Festuca arundinacea*, *Festuca pratensis*, *Lolium perenne*, and *Vulpia myuros* var. *megalura* can also be infected by RBSDV, although the incidence in each species varies (Matsukura *et al*., 2019).

## 3 PROPERTIES OF RBSDV VIRION

**Figure 2:**
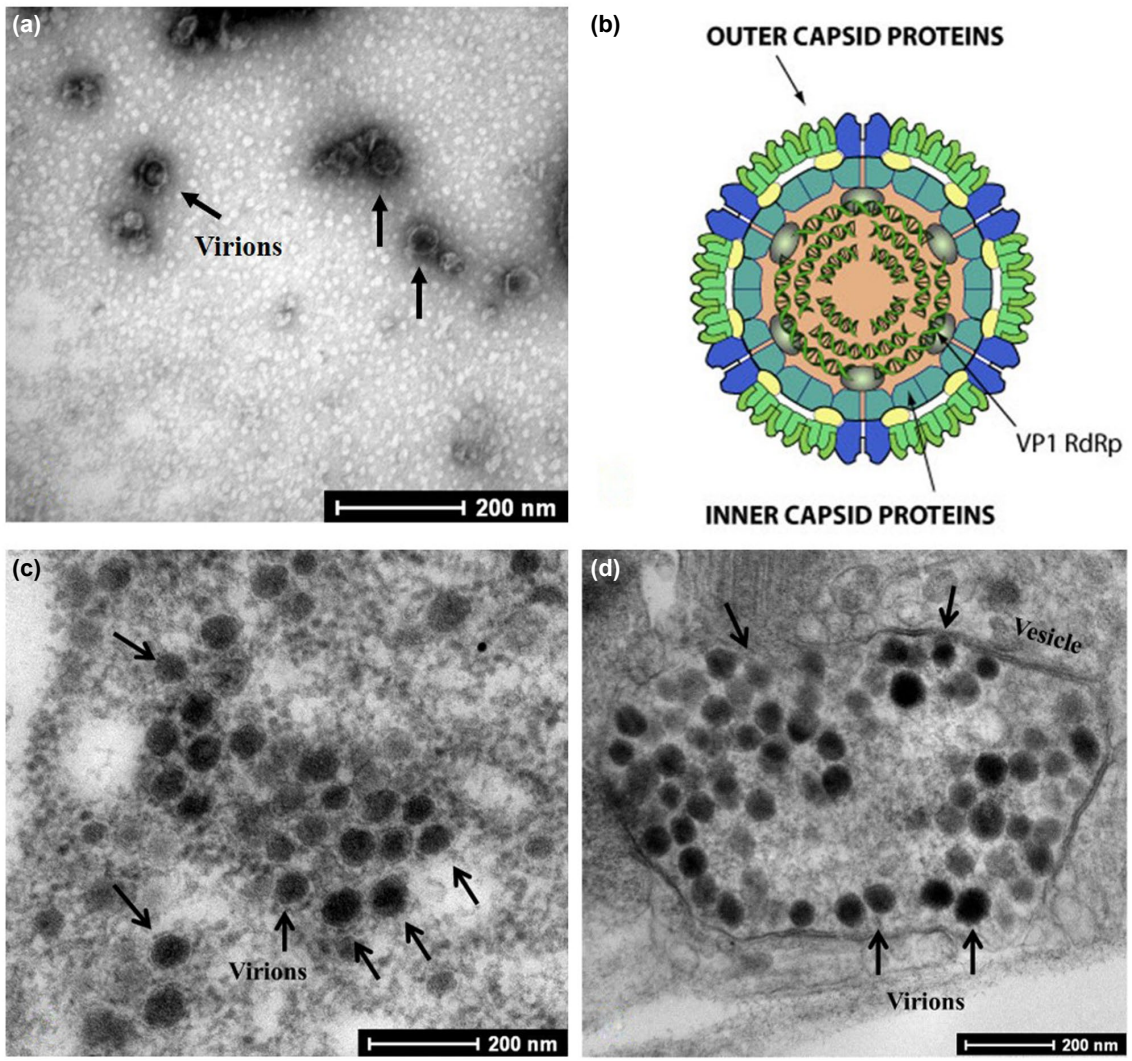
Rice black‐streaked dwarf virus (RBSDV) particles. (a) Transmission electron micrograph (TEM) of purified virus particles. (b) Conceptual diagram of RBSDV particle structure (source: http://viralzone.expasy.org/, Swiss Institute of Bioinformatics). (c) TEM of virus particles in insect midgut samples. (d) TEM of virion‐containing vesicles in insect midgut tissues

Structural and biological features of RBSDV are similar to those of the viruses belonging to the genus *Fijivirus* (Table 1). Virus particles in purified preparations are isometric, c.60 nm in diameter (Figure 2a); the virus particles probably lose their outer projections during purification (Shikata, 1969). Two kinds of particles, c.75–80 nm and c.50–55 nm in diameter, can be observed in cells of infected plants using transmission electron microscopy (Shikata, 1977). Ultrathin sections of diseased leaves reveal inclusions as granular structures (viroplasms) within hypertrophied phloem cells. Small particles of c.50–55 nm occur in the viroplasms, and large particles c.75–80 nm are scattered in the surrounding cytoplasm (Shikata, 1977). Sometimes large particles are arranged within tubular structures or in crystalline aggregates (Shikata, 1969). Virions are not enveloped, but comprise a double‐layered capsid, 50‐nm core with double‐stranded RNAs (dsRNAs), and six proteins (Figure 2b, from http://viralzone.expasy.org/). The outer capsid has a T13 icosahedral symmetry and the inner capsid has a T2 icosahedral symmetry. An RNA‐dependent RNA polymerase (RdRp) in virions is used by the genomic dsRNAs to synthesize mRNAs that are extruded from the virus particles (Coombs, 2008).

**Table 1:** Structural and biological features of viruses in the genus *Fijivirus*

Species names	Virion diameter (nm)	Vector insect	Main host species	Geographical distribution
Fiji disease virus	70	*Perkinsiella saccharicida*, *P. vitiensis*, *P. vastatrix*	*Saccharum officinarum*	Australia, Fiji, Madagascar, Malaysia, New Caledonia, New Hebrides, Philippines, Samoa, Solomon Islands, Thailand, Tonga, Vanuatu
Garlic dwarf virus	60–70	None known	*Allium sativum*	France
Maize rough dwarf virus	70	*Laodelphax striatellus*	*Zea mays*, *Digitaria sanguinalis*, *Echinochloa crus‐galli*	Argentina, China, Czech Republic, France, Germany, Greece, Iran, Israel, Italy, Korea, Norway, Spain, Switzerland, Sweden
Mal de Rio Cuarto virus	60–70	*Delphacodes kuscheli*	*Zea mays*, some species in Poaceae and Cyperaceae	Argentina
Nilaparvata lugens reovirus	80	/	*Nilaparvata lugens* and *Laodelphax striatellus*	Japan
Oat sterile dwarf virus	65–70	*Javesella* spp., *Dicranotropis hamata*	*Avena* spp., *Cynosurus cristatus*, *Hordeum* spp., *Lolium* spp., *Phalaris canariensis*, *Poa annua*, *Secale cereale* and *Triticum* spp.	Czech Republic, Finland, Germany, Norway, Poland, Slovakia, Sweden, UK
Pangola stunt virus	65–70	*Sogatella furcifera*	*Digitaria* spp.	Australia, Bolivia, Brazil, Fiji, French Guiana, Guyana, Malaysia, Peru, Suriname, Taiwan, Venezuela
Rice black streaked dwarf virus	75–80	*Laodelphax striatellus*, *Unkanodes sapporona*, *U. albifascia*	*Oryza sativa, Zea mays, Triticum sativum, Avena sativa, Hordeum vulgare*	China, Japan, Korea
Southern rice black‐streaked dwarf virus	80	*Sogatella furcifera*	*Oryza sativa*, *Zea mays*	China, Japan, Korea, Vietnam

In the vector SBPHs, virus particles accumulate in cells of the fat bodies, salivary glands, and intestines (Shikata, 1969). Large particles are usually scattered in the cytoplasm around the viroplasms or arranged within tubular structures, in crystalline arrays, and in vesicles (Figure 2c). Interestingly, some virion‐containing vesicles can fuse to form a larger vesicle (Figure 2d).

## 4 GENOME ORGANIZATION AND FUNCTIONS

**Figure 3:**
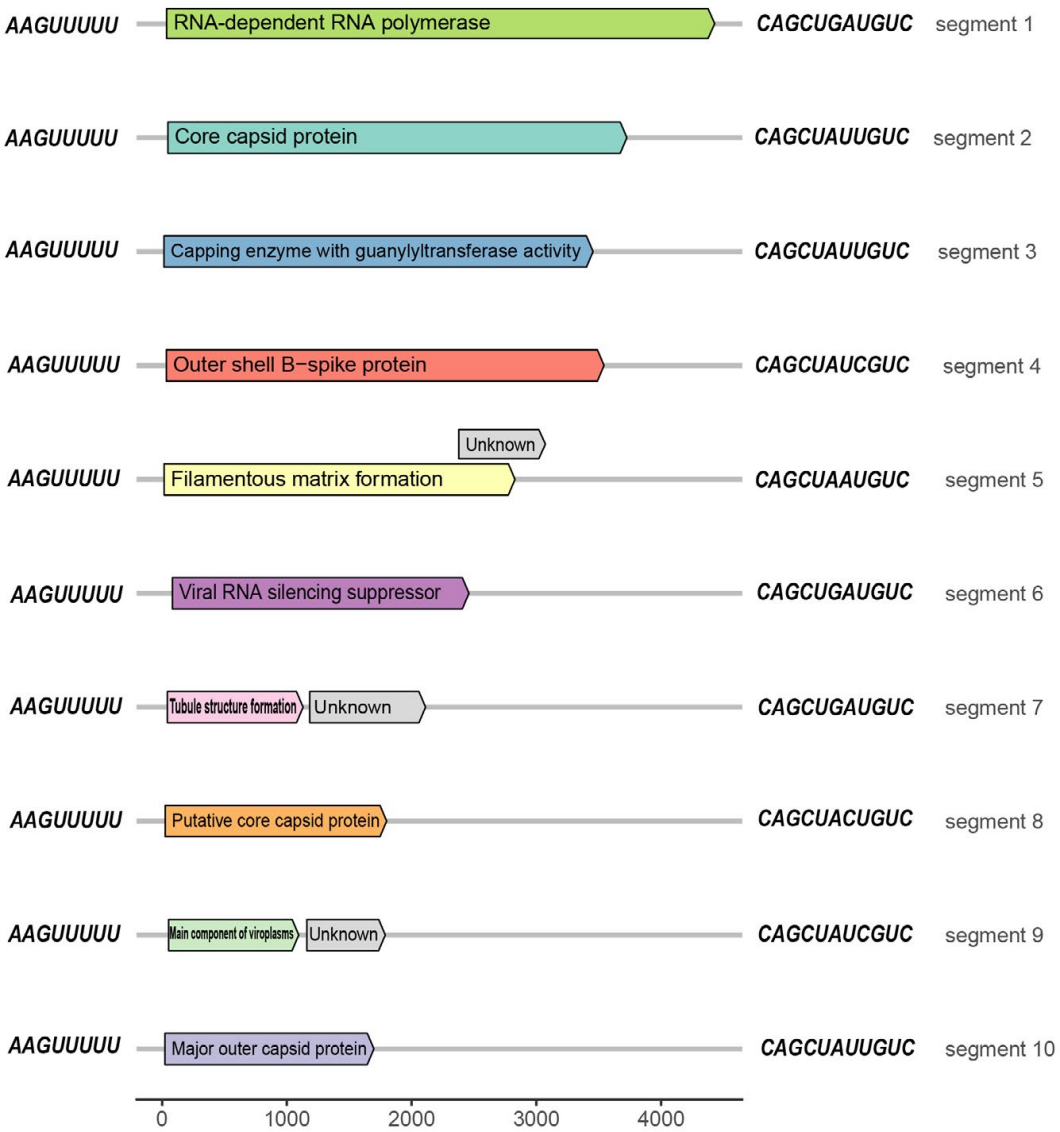
Schematic representation of the organization of rice black‐streaked dwarf virus (RBSDV) genomic RNA segments (linear lines); open reading frames (ORFs) (boxes), and products of each segment

The genome of RBSDV contains 10 dsRNA segments, S1 to S10 in decreasing order of molecular weight (Milne *et al*., 1973, 2005). All RBSDV segments have similar conserved, genus‐specific, terminal genomic sequences of (+) 5′‐AAGUUUUU… CAGCUNNNGUC‐3′, and 7–11 nucleotide inverted repeats adjacent to the terminal conserved sequences (Marzachí *et al*., 1991; Azuhata *et al*., 1992). Most RBSDV genomic segments contain one open reading frame (ORF), but S5, S7, and S9 contain two ORFs (Azuhata *et al*., 1993; Isogai *et al*., 1998a; Firth and Atkins, 2009). As for the counterpart viruses that infect vertebrates and insects, individual mRNAs are thought to be transcribed at the same time from the minus strands of the dsRNA segments by RdRp and other cofactors in virus particles (Coombs, 2008). Early in infection, RdRp begins transcription from each of the genomic dsRNAs, then the mRNAs are extruded through channels at the 5‐fold axis around the cores. Early mRNAs are translated in the cytoplasm of the host cell and the accumulating proteins begin to form viroplasms or function biologically (Wei and Li, 2016). The largest segment S1, 4,501 nucleotides (nt) long, encodes RdRp (P1, 168.8 kDa) (Wang *et al*., 2003). S2, 3,812 nt long, encodes the major core structural protein (P2, 141.5 kDa) (Zhang *et al*., 2001; Wang *et al*., 2003). S3, 3,572 nt long, encodes a protein with some guanylyltransferase activity (P3, 132.0 kDa) (Supyani *et al*., 2007). S4, 3,617 nt long, encodes an outer‐shell B‐spike protein (P4, 135.6 kDa) (Zhang *et al*., 2001). S5, 3,164 nt long, has a major ORF that is partially overlapped by a second ORF but in a different reading frame and thus encodes two proteins, P5‐1 (106 kDa) and P5‐2 (27 kDa) (Yang *et al*., 2014). P5‐1 is a viroplasm component (Xie *et al*., 2017), and P5‐2 might be functionally and evolutionarily related to the C‐terminal part of Fiji disease virus P5 (Yang *et al*., 2014). S6, 2,645 nt long, encodes a viral RNA‐silencing suppressor (P6, 89.9 kDa) (Zhang *et al*., 2005). P6 protein has an intrinsic ability to self‐interact and forms viroplasm‐like structures (VLSs) without other RBSDV proteins or RNAs (Wang *et al*., 2011). P6 recruits P9‐1 to the VLSs by direct protein–protein interaction, suggesting it may be involved in viroplasm nucleation and virus morphogenesis (Sun *et al*., 2013). The first ORF of 2,193 nt S7 encodes nonstructural protein P7‐1 (41.0 kDa), which is involved in forming the structural matrix of the tubular structures in infected tissues (Liu *et al*., 2011). In addition, P7‐1 can use the endoplasmic reticulum (ER)‐to‐Golgi secretory pathway and the actomyosin motility system for virus intracellular transport (Sun *et al*., 2013c). P7‐2 (309 amino acids long), encoded by the second ORF of S7, can interact with different SKP1 proteins, an important component of SCF (SKP1/Cullin1/F‐box protein/Rbx1) E3 ubiquitin ligase, but the biological implication is unknown (Wang *et al*., 2013). Proteins encoded by S8 (1,936 nt long) and S10 (1,801 nt long) are the components of the major capsid (P8, 68.1 kDa) and outer capsid (P10, 63.1 kDa), respectively (Isogai *et al*., 1998b; Liu *et al*., 2007b). P8 is also targeted to the nucleus of insect and plant cells via its N‐terminal 1–40 amino acids and possesses potent active transcriptional repression activity, suggesting that it probably enters the nucleus of the host cell where it acts as a negative transcriptional regulator of host gene expression (Liu *et al*., 2007b). P10 might modulate the ER stress response and the unfolded protein response to facilitate capsid assembly to increase the ER folding capacity (Liu *et al*., 2007a; Sun *et al*., 2013c). S9, 1,900 nt long, encodes the main component of viroplasms in infected cells, P9‐1 (39.9 kDa), which is involved in viral replication (Akita *et al*., 2012) and directly interacts with P6 and recruits it into viroplasms (Zhang *et al*., 2008). It indirectly recruits P5 into the viroplasms through the association of P6 and P5 (Sun *et al*., 2013b). In addition, P9‐1 is present as dimers, tetramers, and octamers in vitro, but only the octamer is an important form and gradually binds to ssRNA to form a saturated complex (Wu *et al*., 2013a). The function of P9‐2 (24.2 kDa) is unknown. The length of the 10 segments and the functions of their encoded proteins are shown in Figure 3. The whole genome information can be obtained from GenBank assembly accession number GCA_000852945.1.

The most recent studies have revealed new functions of RBSDV‐encoded proteins. P5‐1 inhibits the ubiquitination activity of SCF E3 ligases through an interaction with OsCSN5A, a subunit of the COP9 signalosome complex in rice, and hinders the RUBylation/deRUBylation of cullin‐ring ligases CUL1, leading to an inhibition of the jasmonate response pathway and an enhancement of virus infection in rice (He *et al*., 2020). The expression of P10 in plants alleviates the symptoms caused by RBSDV and the closely related SRBSDV, but renders the plants more susceptible to the unrelated RSV. Transcriptomic data reveal that the expression of P10 in plants significantly suppresses the expression of rice‐defence related genes, which may play important roles in resistance to RSV infection (Zhang *et al*., 2019). During the virus–vector insect interaction in vivo, the N‐terminus region of P10 is responsible for impairing the innate immunity of *L. striatellus* to increase virus replication, and this function is achieved through altering the subcellular location of the receptor for activated protein kinase C1 and modulating the structure and function of the protein kinase C activity in its vector (Lu *et al*., 2019).

Genetic variation and recombination have been characterized in each or all segments of the RBSDV genome. Phylogenetic analysis of each or all segments of rice and maize isolates mainly from China showed that they could be clustered into two groups regardless of host or geographical origin (Zhou *et al*., 2015b, 2018). An analysis of diversification and selection of the entire genome determined low codon adaptation index values ranging from 0.1878 to 0.2918 (Zhou *et al*., 2017). Nucleotide sequence diversity analysis indicated that the structural proteins of RBSDV, such as P2 and P4, are all more conserved than nonstructural proteins such as P9‐1. Numerous recombinants have been found in S8, S9, and S10, but no recombination events have been detected between RBSDV and SRBSDV (Li *et al*., 2012; Zhou *et al*., 2015b, 2017). The nucleotide sequence identities among the S8 of RBSDV and SRBSDV isolates ranged from 66.2% to 68.2%, considerably lower than those within RBSDV (94.1%–99.9%) or SRBSDV (93.9%–100%) isolates. Most S8 polymorphisms were identified in the region from 1,000 to 1,200 bp in RBSDV and in the region from 500 to 700 bp in SRBSDV (Zhou *et al*., 2017).

## 5 DIAGNOSIS

As mentioned above, RBSDV generally induces extreme dwarfing, darkened leaves, and white waxy enations in the host plants, but these symptoms are not very useful for distinguishing RBSDV from other viruses in the field, especially SRBSDV. In most cases, immuno‐ or molecular diagnostic aids can be used. For immunodiagnosis, three monoclonal antibodies (MAbs) for RBSDV detection in field plants and vector insects have been produced using crude extracts from white waxy enations (tumours) of RBSDV‐infected maize as the immunogen (Wu *et al*., 2013b). These MAbs reacted with crude extracts from RBSDV‐infected plant tissues and viruliferous insects, but not with rice plants infected with rice dwarf virus, SRBSDV, rice ragged stunt virus, and RSV. Unquestionably, they have no reaction with healthy plant tissues and nonviruliferous vectors. Three serological assays, antigen‐coated‐plate enzyme‐linked immunosorbent assay (ELISA), dot ELISA, and colloidal gold immunochromatographic strips based on RBSDV‐specific MAbs, are widely used to detect the virus in rice, maize, wheat, and vector insect samples in China (Wu *et al*., 2013b). Molecular diagnostics based on reverse transcription (RT)‐PCR, quantitative real‐time RT‐PCR, and RT‐loop‐mediated isothermal amplification for RBSDV detection and diagnosis have also been developed in China (Zhang *et al*., 2013; Li *et al*., 2015; Hajano *et al*., 2015; Du *et al*., 2019). Moreover, a one‐step multiplex RT‐PCR for the simultaneous detection of three rice viruses in Korea and four wheat and rice viruses in China has been developed (Cho *et al*., 2013; Zhang *et al*., 2017).

## 6 VIRUS TRANSMISSION

*L. striatellus*, *Unkanodes sapporona*, and *U. albifascia* are known to transmit RBSDV in a persistent propagative nontransovarial manner, but *L. striatellus* is the main vector because of its high density in the field (Kuribayashi and Shinkai, 1952; Shinkai, 1962). SBPHs can acquire RBSDV after a minimum acquisition access period of 30 min, but 1 day is the optimal period (Hajano *et al*., 2015). However, the length of the acquisition period also depends on the nymphal stage of the insect; the first instar can acquire the virus more effectively compared to other stages. The inoculation access period ranges from 7 to 35 days (in most cases, 7–21 days) (Ou, 1985), with a 5‐min minimum inoculation feeding, but in 1–3 hr 50% of infected insects can transmit RBSDV to healthy plants, and a longer time is associated with higher transmission; after acquisition, the insects remain viruliferous until death (Shinkai, 1962). Generally, acquisition and transmission efficiencies are relatively high; the 55th generation of laboratory‐reared insects still retain efficiencies of 68.24% and 31.22%, respectively (Yang *et al*., 2017). The insects even acquired RBSDV from infected rice leaves that had been frozen then thawed and cut into 3–4 cm long pieces and stored in wet cotton gauze for at least 4 hr in a conical flask until the leaves had completely rehydrated, then transmited the virus to healthy rice and maize plants (Li *et al*., 2011). In a quantitative comparison of the spatial distribution of RBSDV titres in its vector insects using quantitative real‐time RT‐PCR, RBSDV genome equivalent copies/ng total RNA increased over time in the whole body, the transmission efficiency of RBSDV was significantly positively correlated with genome equivalent copies in the salivary glands (Hajano *et al*., 2015).

## 7 DISEASE CYCLE AND EPIDEMIOLOGY

**Figure 4:**
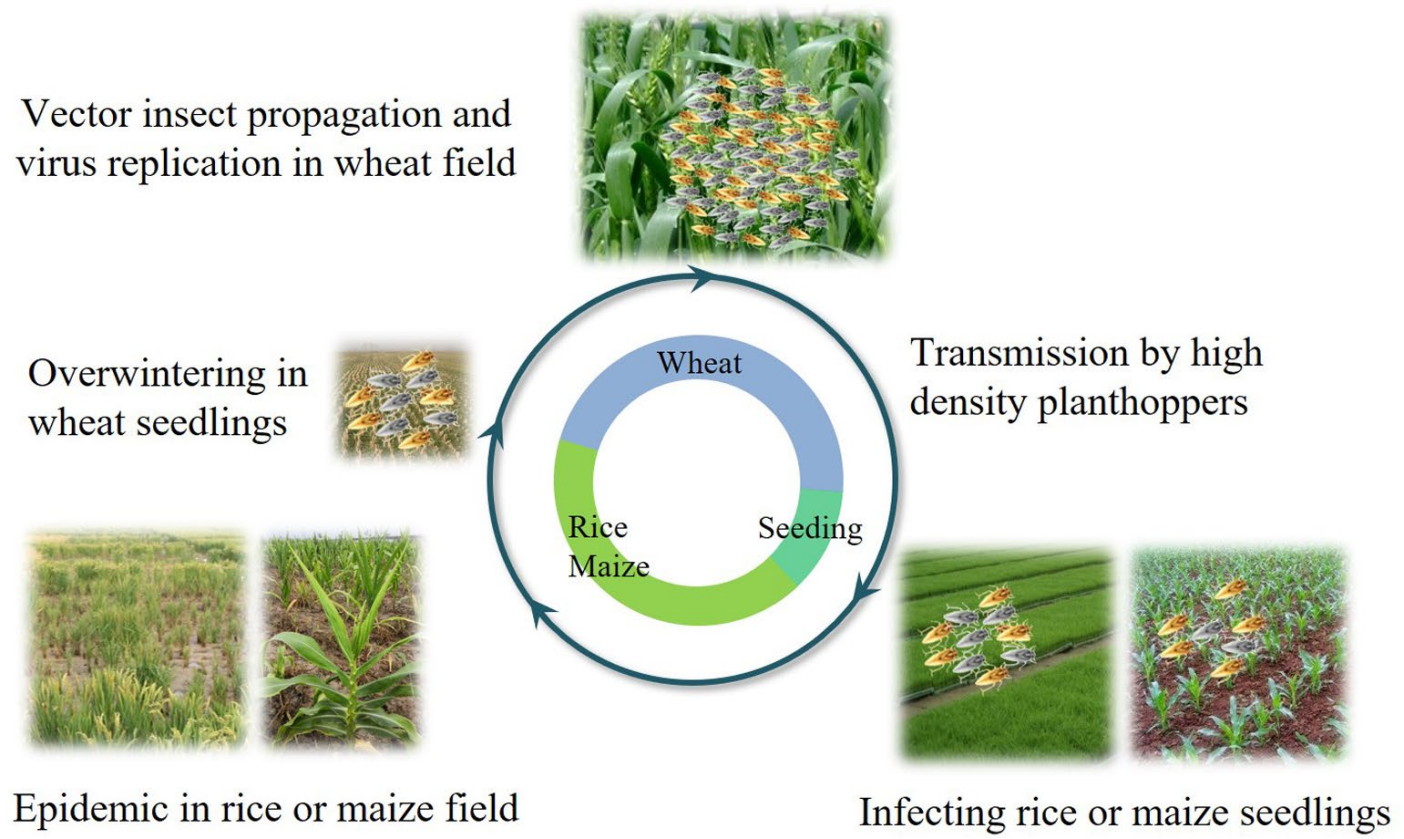
The infection cycle of rice black‐streaked dwarf virus (RBSDV) induced diseases on three cereal crops (rice, maize, and wheat). The virus can be transmitted among its different hosts by *Laodelphax striatellus*, which mainly overwinters and propagates in wheat fields, resulting in disease outbreaks on rice and maize

For epidemics of RBSDV‐induced cereal diseases, migration and virus transmission of SBPHs among the different crops or gramineous weeds are essential because the vector insect is the only means of virus spread in nature (Hogenhout *et al*., 2008; Wei and Li, 2016). Overwintering SBPHs play an important role in carrying virus particles from one host to another or from one season to the next and may infect cereal crops in the spring and early summer at lower levels, but infection gradually increases in later generations to cause heavy epidemics and yield losses under certain conditions (Ren *et al*., 2016). In most regions with epidemics in China, the infection cycle can be described as follows: RBSDV and its vector SBPHs overwinter in wheat, barley, and some gramineous weeds, including *Alopecurus aequalis*, *Beckmannia syzinachne*, and *Poa annua*. In the early spring, the overwintered nymphs begin to develop into adults to lay eggs on the overwintered host plants (mainly wheat), then acquire the virus by feeding on infected overwintered host plants. They develop into adults, migrate to rice and maize seedlings or other gramineous weeds to feed and lay eggs, and transmit the virus in the newly colonized areas in late May and early June. In most cases, each successfully mated female lays 150–200 eggs on infected seedlings. The newly hatched nymphs become viruliferous then disperse into other fields, resulting in new disease outbreaks. After mid‐October, nymphs migrate to wheat, barley, and some gramineous weed species for overwintering and can transmit the virus to these hosts again, thus finishing the infection cycle (Figure 4).

Based on the virus infection cycle, we conclude that the large area planted with susceptible varieties is a primary reason for the disease, but efficient transmission of the virus by the vector SBPHs is the direct reason for disease epidemics based on the data obtained in the last decades. Abiotic factors such as cultivation system, improper use of pesticides, and climatic conditions also contribute to disease epidemics by affecting the development of the vector insects and their population dynamics. As areas planted with susceptible hybrid rice varieties Xieyou 46 and Xianyou 10 increased in the early 1990s, the incidence of RBSDV increased year after year in Zhejiang Province of China, and an epidemic broke out in 1996 and 1997 (Chen and Zhang, 2005). Since 2004, several japonica rice varieties, such as Huaidao 5, with resistance to RSV but high susceptibility to RBSDV, have been planted in Jiangsu Province of China. As rice stripe disease decreased, black‐streaked dwarf disease increased rapidly (Zhu *et al*., 2015). We evaluated the resistance of 66 japonica and indica rice varieties, grown commercially on a large scale in China in recent years. In the greenhouse, only seven varieties responded as moderately resistant, 47 as moderately susceptible, and 12 as highly susceptible. However, in the field 13 varieties were moderately resistant, 44 moderately susceptible, and nine highly susceptible. Only a few varieties, such as Liandao 9805, YLiangyou 3399, and Liangyou 1129, were visually assessed as moderately resistant in both the greenhouse and the field (Hajano *et al*., 2016). Thus, the widespread use of susceptible varieties provided the setting for RBDSV outbreaks in the rice districts along the Yellow River of China during 2013–2014.

The first outbreak of RBSDV in Zhejiang Province of China during 1963 and 1966 was attributed to a change from single rice cultivation to a double‐cropping system with early‐ and late‐season rice. In the double‐cropping rice system, SBPHs might generate two generations on early season rice, and the population can increase dozens of times then migrate and transmit virus to late‐season rice, resulting in large RBSDV outbreaks (Chen, 1966). In the late 1960s, the cultivation system in the same region changed to a three‐crop system (green manure–early‐season rice–late‐season rice), and the SBPH population could be naturally managed by the harvest and transplanting process of the system. However, in the late 1980s, a wheat or early‐season rice–late season rice system gradually became favoured in this region and RBSDV epidemics developed again in the early 1990s (Chen *et al*., 1993). During the last three decades, a double‐cropping system for either rice and wheat or maize and wheat was widely implemented in central and eastern China. In these cropping systems, hosts for SBPHs are abundant, and population density and overwintering insects increased (Figure S2). Moreover, the virus was transmitted among its different hosts, resulting in outbreaks of rice stripe, black‐streaked dwarf, and maize rough dwarf diseases in these regions (Ren *et al*., 2016). On the other hand, improper application of pesticides might also affect the development and population dynamics of vector insects. For example, triazophos was usually used to control *Chilo suppressalis* on early‐season rice in Zhejiang Province of China in the 1990s, but it can stimulate oviposition of SBPH females and increase the population density later on early‐season rice and thus the opportunities for virus transmission (Chen *et al*., 2000).

## 8 DISEASE MANAGEMENT

Integrated disease management seeks to reduce the impact of the disease in regions with epidemics (Hogenhout *et al*., 2008; Wei and Li, 2016). The critical aspect of such a strategy is to halt the cycle of virus transmission by the vector SBPHs in the absence of commercial varieties with resistance to RBSDV. The most economical and effective preventive measure, covering rice seedling nurseries with insect‐proof nets, has been widely used in central and eastern China to control rice stripe and black‐streaked dwarf diseases (Sun *et al*., 2013a; Ren *et al*., 2016). For chemical control of the vector SBPHs, pesticides should first be applied in wheat fields to control the overwintering or the first generation of insects and in rice seedling nurseries to decrease the SBPH population and thus transmission (Ren *et al*., 2016). In regions with a high‐density viruliferous population of SPBHs, the overwintering or the first generation of SBPHs should be controlled with pesticides such as pymetrozine, imidacloprid, nitenpyram, thiamethoxam, or buprofezin (Sun *et al*., 2013a). To control immigrant SBPHs in rice seedling nurseries, seed coating with pesticide is recommended before sowing, then seedlings should receive pesticide 3–5 days before transplanting (Sun *et al*., 2013a; Ren *et al*., 2016). Chemical control in the field is not suggested except when a heavy epidemic is forecasted, and different kinds of pesticides should be applied in fields over time during the season to avoid or delay the development of resistance in the SBPHs. Changes in the cultivation system can also control RBSDV epidemics by affecting the SBPH life cycle. During the late 1960s, RBSDV epidemics were effectively controlled in eastern China by decreased use of the wheat–rice rotation and increased use of the green manure–rice system, or wheat was changed to various vegetable crops or double‐cropping with rice was changed to single rice cropping in the 1990s (Chen and Zhang, 2005). To control RBSDV in maize, three measures have been carried out in northern China. (a) The first generation of SBPHs in wheat fields is controlled and maize seedlings are protected by chemicals. (b) The sowing time is adjusted so that the first generation of SBPHs migrating from wheat fields does not coincide with the susceptible growth period of maize (before seedlings have three to five leaves), that is, spring maize should be sown as early as April, but the sowing time of summer maize should be postponed until the middle of June. (c) Decreased use of interplanting maize with wheat, or maize seed dressed with suitable pesticide, should be recommended in the epidemic regions (Chen and Zhang, 2005; Sun *et al*., 2013a).

Although commercial varieties lack resistance to RBSDV, several resistance sources have been identified in the germplasm of diverse cultivars of rice and maize and related species (Luan *et al*., 2012; Hajano *et al*., 2016; Feng *et al*., 2019). These studies revealed a low percentage of resistant lines and no immune or highly resistant materials among the temperate sources of japonica germplasm, but several aus‐type, indica, and tropical japonica subspecies have high levels of resistance, which have been reported to be controlled by quantitative trait loci (QTLs) or multiple genes (Wang *et al*., 2010; Zheng *et al*., 2012). To facilitate the use and transfer of host resistance in breeding programmes, recent QTL mapping or genome‐wide association studies have identified genomic regions associated with RBSDV resistance and linked DNA markers (Feng *et al*., 2019; Xiao *et al*., 2019). Several QTLs on different chromosomes have been identified in japonica cultivar Koshihikari, and indica cultivars Minghui 63, WR24, Tetep, IR36, and 9194 (Wang *et al*., 2010; Li *et al*., 2013; Zhou *et al*., 2015a; Zhang *et al*., 2016; Sun *et al*., 2017; Xu *et al*., 2018). Some QTLs such as qRBSDVD5, qRBSDVD6, and qRBSDV‐6.3 that confer stable resistance have been introgressed into susceptible rice cultivars by marker‐assisted selection (Feng *et al*., 2019; Xiao *et al*., 2019). In addition, five QTLs, qMRD2, qMRD6, qMRD7, qMRD8, and qMRD10, were identified in elite maize line 90110. On chromosome 8, qMRD8 proved to be a major QTL conferring resistance that explained 12.0%–28.9% of the phenotypic variance for disease severity (Luan *et al*., 2012).

Some studies have focused on resistance or susceptibility mechanisms by analysing endogenous phytohormones, which are severely disturbed by RBSDV. The levels of abscisic acid and cytokinins increase, while indole‐3‐acetic acid, gibberellins, jasmonic acid (JA), and salicylic acid decrease after RBSDV infection (Huang *et al*., 2018; Liu *et al*., 2020, 2020b). JA‐mediated defence can suppress the brassinosteroid‐mediated susceptibility to RBSDV infection through efficient suppression of the expression of brassinosteroid pathway genes, and this inhibition depends on the JA coreceptor OsCOI1 (He *et al*., 2017). Brassinosteroid‐mediated susceptibility is involved in the inhibition of the JA/salicylic acid‐mediated plant defence response and the reduction of peroxidase‐mediated accumulation of reactive oxygen species (Zhang *et al*., 2019). The abscisic acid pathway might play a negative role in rice defence against RBSDV by suppressing the JA pathway and regulating the reactive oxygen species level (Xie *et al*., 2018). In addition, genomic analysis indicated that the expression of three nonconserved and 28 conserved miRNAs are significantly altered in response to RBSDV in maize. Two negatively regulated genes, GRMZM2G069316 and GRMZM2G031169, which are the target genes for miR169i‐p5 and miR8155, were identified as encoding a nucleolin and a NAD(P)‐binding Rossmann‐fold superfamily protein in maize, respectively (Zhou *et al*., 2016).

Transgenic rice lines harbouring a hairpin RNA (hpRNA) construct targeting the P7‐2 or P8 genes of RBSDV induce a high‐level resistance in rice against the disease (Ahmed *et al*., 2017). Transgenic rice lines that harbour an hpRNA construct targeting the P1, P2, P6, and P10 genes also have strong resistance to RBSDV infection. The hpRNA transgene was expressed in the highly resistant transgenic lines, giving rise to abundant levels of 21–24‐nt small interfering RNAs (Wang *et al*., 2016). Additionally, three dimeric artificial microRNA precursor expression vectors (pamiR‐M, pamiR‐3, and pamiR‐U) that simultaneously target the structural genes of RSV and RBSDV were inserted into rice, and transgenic lines with pamiR‐U, which targeted the 3′ UTR region induced higher virus resistance: 54.17% against RSV and 45.83% against RBSDV (Sun *et al*., 2016).

## 9 FUTURE PROSPECTS

In recent decades, several epidemics of cereal diseases caused by RBSDV have significantly reduced yield and quality. As expected, biotic and abiotic factors are associated with these intermittent epidemics, but the underlying reasons await further research. By continuously monitoring the disease system in the rice districts along the Yellow River of China in recent years, we have found that even though the population of viruliferous insects was high enough and the growth period of susceptible hosts coincided with the migration time of the first generation of SBPHs from wheat fields, the disease has been decreasing in rice and maize since its peak in 2013–2014. It has therefore been very difficult to predict when and where an outbreak will arise. To better understand the complex disease system and provide an accurate forecast to guide control measures, we believe that future research should focus on, but not be limited to, the following areas.

First, an effective management strategy relies on a good understanding of the biology of the disease cycle. Because the vector insect is the only natural means of virus spread, efficient transmission of vector SBPHs is the direct cause of epidemics. A better understanding of the transmission mechanism will help dissect the cause of the intermittent epidemics. Many areas need to be addressed, including (a) how does the virus overcome barriers to transmission in insects by interacting with insect components to achieve highly efficient transmission, (b) epigenetic methylation of genes of viruses and insects, which may affect interactions between the virus and vector insect, (c) mutually beneficial or antagonistic mechanisms among viruses transmitted by SBPHs, and (d) impact of gut microbiota in vector insects on the transmission. Some of this research work has been done for other virus–vector insect combinations (Wei and Li, 2016; Qin *et al*., 2018; Wang *et al*., 2019a,b; Liu *et al*., 2020, 2020a), but the results for these systems need to be confirmed and compared with those for RBSDV and SBPHs.

Second, because genetic resistance for disease control is the most sustainable and environmentally friendly solution, plant pathologists and breeders should use currently identified resistant germplasms and search for novel sources of resistance. Research is needed to investigate the genetics of resistance in the identified rice and maize cultivars and breeding lines, and find more DNA markers linked to the resistance genes or QTLs. As soon as possible the known QTLs should be used in breeding programmes to quickly incorporate resistance into local cultivars. Although it could take a long time to transfer the resistance genes from wild or indica rice to japonica cultivars, it is worth doing because these genes could confer high levels of resistance to RBSDV.

Third, a better understanding of the interactions between the host, RBSDV, and the vector insect is needed to develop rice or maize cultivars with durable resistance and illuminate the deeper reasons for the intermittent nature of the epidemics. Next‐generation sequencing, comparative genomics, and transformation techniques can serve as powerful tools to identify host genes involved in resistance and susceptibility and host‐range determinants. The susceptibility genes in the host that either directly or indirectly interact with virus genes can be subsequently identified, then altered using gene‐editing technologies.

Last and more immediately, real‐time monitoring of viruliferous SBPHs and an accurate forecasting system are the bases for making an effective management strategy. To accomplish this purpose, relevant research institutions should strengthen cooperation with agricultural extension services which can develop a sound, evidence‐based forecasting system based on the data obtained by research institutions. Moreover, effective management strategies must be carried out by farmers. The researchers should give concise guidance for the farmers to prevent and control disease outbreaks in the fields.

## Data Availability

Data sharing is not applicable to this article as no new data were created or analysed in this study.
