# Supplementary material for: Rice black‐streaked dwarf virus: From multiparty interactions among plant–virus–vector to intermittent epidemics
Source: Mol Plant Pathol. 2020 Jun 8;21(8):1007–19. doi: 10.1111/mpp.12946 (PMC7368121; doi:10.1111/mpp.12946)
Supplement: Supplementary file 1 [file MPP-21-1007-s001.docx]

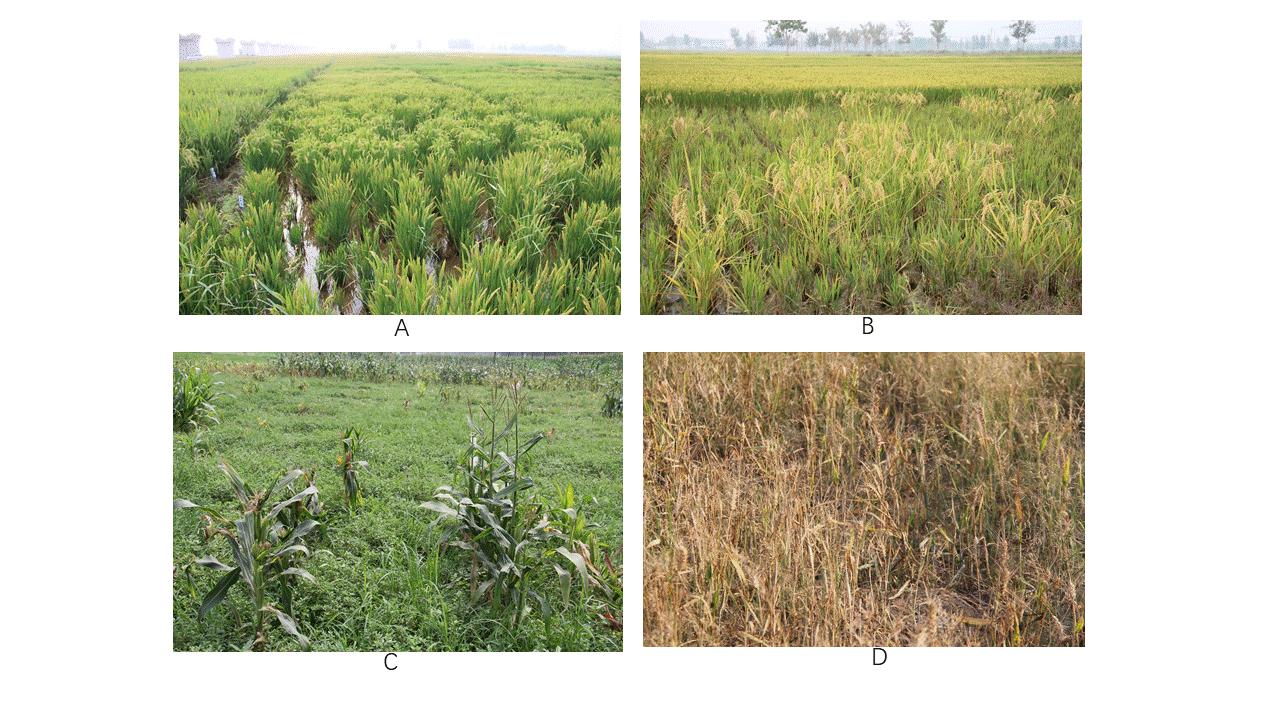


Fig. S1 Fields with severe disease caused by rice black-streaked dwarf virus (RBSDV). A and B: Rice, C: maize, D: wheat.
