# Supplementary material for: Rice black‐streaked dwarf virus: From multiparty interactions among plant–virus–vector to intermittent epidemics
Source: Mol Plant Pathol. 2020 Jun 8;21(8):1007–19. doi: 10.1111/mpp.12946 (PMC7368121; doi:10.1111/mpp.12946)
Supplement: Supplementary file 2 [file MPP-21-1007-s002.docx]

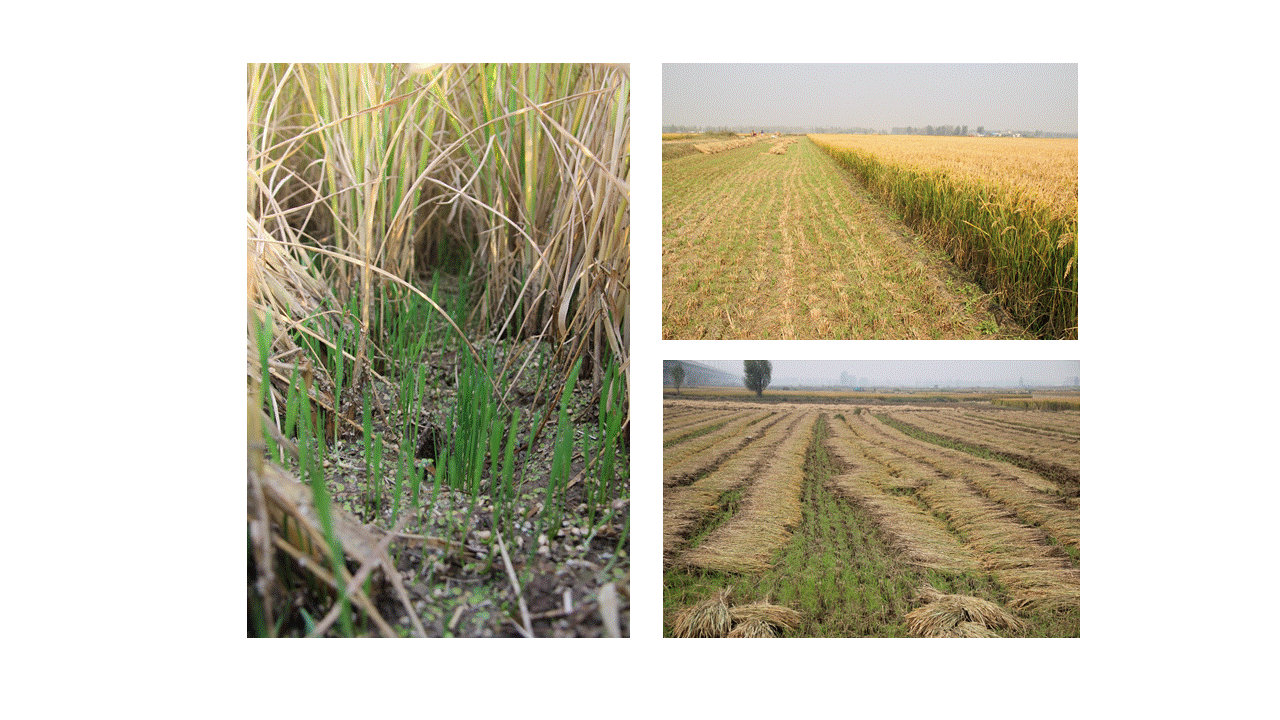


Fig. S2 Double-cropping system with rice and wheat rotation showing rice and wheat coexisting for a long time in the autumn. In this system, abundant hosts are available for insects of *Laodelphax striatellus*, which then increase in population density and overwintering nymphs. The virus is transmitted among its different hosts, resulting in outbreaks of RBSDV-induced diseases in rice, maize and wheat.
